# The Conserved *Dcw* Gene Cluster of *R*. *sphaeroides* Is Preceded by an Uncommonly Extended 5’ Leader Featuring the sRNA UpsM

**DOI:** 10.1371/journal.pone.0165694

**Published:** 2016-11-01

**Authors:** Lennart Weber, Clemens Thoelken, Marcel Volk, Bernhard Remes, Marcus Lechner, Gabriele Klug

**Affiliations:** 1 Institute of Microbiology and Molecular Biology, IFZ, Justus-Liebig-University Giessen, Giessen, Germany; 2 Institute of Pharmaceutical Chemistry, Philipps-University Marburg, Marburg, Germany; East Carolina University Brody School of Medicine, UNITED STATES

## Abstract

Cell division and cell wall synthesis mechanisms are similarly conserved among bacteria. Consequently some bacterial species have comparable sets of genes organized in the *dcw (**d**ivision and* *c**ell* *w**all)* gene cluster. *Dcw* genes, their regulation and their relative order within the cluster are outstandingly conserved among rod shaped and gram negative bacteria to ensure an efficient coordination of growth and division. A well studied representative is the *dcw* gene cluster of *E*. *coli*. The first promoter of the gene cluster (mraZ1p) gives rise to polycistronic transcripts containing a 38 nt long 5’ UTR followed by the first gene *mraZ*. Despite reported conservation we present evidence for a much longer 5’ UTR in the gram negative and rod shaped bacterium *Rhodobacter sphaeroides* and in the family of *Rhodobacteraceae*. This extended 268 nt long 5’ UTR comprises a Rho independent terminator, which in case of termination gives rise to a non-coding RNA (UpsM). This sRNA is conditionally cleaved by RNase E under stress conditions in an Hfq- and very likely target mRNA-dependent manner, implying its function in *trans*. These results raise the question for the regulatory function of this extended 5’ UTR. It might represent the rarely described case of a *trans* acting sRNA derived from a riboswitch with exclusive presence in the family of *Rhodobacteraceae*.

## Introduction

There are only rare cases for highly conserved gene clusters throughout bacterial genomes due to evolutionary dynamics. Examples for such clusters are genes for ribosomal proteins, the *atp* operon or the *dcw (division and cell wall)* gene cluster [1]. However, conservation of *dcw* genes, their regulation and especially their arrangement within the cluster are outstandingly conserved within bacterial groups of similar taxon and cell shape [2]. Besides regulatory mechanisms the conserved order of the genes may ensure an efficient coordination of growth and division as assumed by the *genomic channeling hypothesis* [3]. A well described example for such conservation is the *dcw* gene cluster of gram negative and rod shaped bacteria.

Dcw gene regulation was studied intensively in *E coli*, but is not fully understood due to numerous regulatory features like internal promotors, transcript stabilities and protein ratios. It is well known that the first promoter (*mraZ*1*p*) of the gene cluster in *E*. *coli* (16 genes in total) gives rise to polycistronic transcripts containing a 38nt long 5’ UTR followed by the first gene *mraZ* [4]. Downstream of *mraZ* transcription can potentially continue up to the last gene of the locus (*envA*) that harbors a Rho independent terminator [5].

Here we present evidence for a much longer 5’ UTR in the gram negative and rod shaped bacterium *Rhodobacter sphaeroides* also present in other members of *Rhodobacteraceae*. In *R*. *sphaeroides* this 268 nt long 5’ UTR features a Rho independent terminator 84 nt upstream of *mraZ*, which in case of transcriptional termination gives rise to a non-coding RNA of 206 nt length. This transcript was described as an orphan sRNA named RSs0682 [6], henceforth renamed UpsM (upstream sRNA of *m**raZ*). Here we also show that conditional processing of UpsM requires the RNA chaperon Hfq, the endoribonuclease RNase E and induction of the RpoHI/II regulon.

Our results raise the question for the complex regulatory function of this extended 5’ UTR, which might represent a rare dual function riboswitch exclusively present in the family of *Rhodobacteraceae*.

## Results

### dRNA-seq Hints at an Extended 5’ UTR of the *Dcw* Gene Cluster of *R*. *sphaeroides*

**Fig 1:**
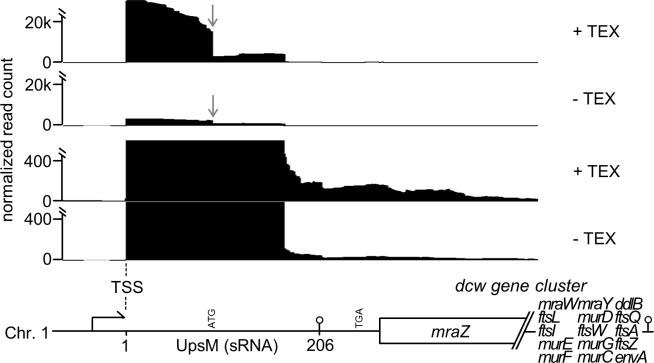
dRNA-seq shows a long 5’ UTR of the *mraZ* gene in *R*. *sphaeroides*. Modified screenshots taken from IGB (integrated genome browser) visualizing the coverage at the genetic locus of *mraZ*. Shown are normalized cDNA reads on a large scale (upper two panels) and a smaller scale (lower two panels) obtained from TEX treated and untreated total RNA isolated from an exponentially and microaerobically grown *R*. *sphaeroides* 2.4.1 culture. The genetic context is displayed at the bottom. *mraZ* is the first gene of the *dcw* gene cluster. Position 1 reflects the TSS of sRNA UpsM (206 nt) 268 nucleotides upstream of *mraZ*. The terminator of UpsM is indicated as hairpin structure and a processing site within the sRNA is highlighted by an arrow.

The sRNA UpsM (previously RSs0682) was described as the most abundant orphan sRNA of *R*. *sphaeroides*, which is encoded in the intergenic region (IGR) upstream of *mraZ*, the first gene of the *dcw* (division and cell wall) gene cluster. A Rho-independent terminator was predicted at the 3’ end of the sRNA locus. Identification of UpsM was based on deep sequencing of cDNA libraries using 454 pyrosequencing. The coverage of UpsM was comparably low (~2,000 reads per library) in the initial sequencing study [6]. Since sequencing technologies have rapidly evolved and nowadays generate millions of reads concomitant with visualisation of transcripts with low abundance, we re-analysed the UpsM locus in a dRNA-seq dataset that has been generated using Illumina sequencing technology (Fig 1 and S1 Fig) without and with prior TEX (terminator-5´phosphate dependent exonuclease) treatment of the RNA to enrich primary transcripts. This confirmed the presence and high abundance of UpsM and the transcriptional start site (TSS). In contrast to the low-coverage 454 pyrosequencing study, additional observations were possible. First, the processing site within UpsM, which was already detected by Northern blot analysis and 5’ RACE [6], becomes apparent by a sudden decrease of reads especially after TEX treatment. Secondly, the downstream gene *mraZ* is not preceded by a separate TSS. This is surprising since *mraZ* is the first gene of the *dcw* gene cluster. Therefore it should be expressed in exponentially growing cells in the course of cell wall synthesis and cell division as described for other bacteria [4, 7–11]. This observation led to the following assumptions: *MraZ* transcription depends on the UpsM promoter and there is no additional promoter/TSS exclusively present for *mraZ*. If this is true, the terminator of UpsM has to allow read-throughs in order to guarantee transcription of *mraZ*. This has several consequences: 1.) *mraZ* or the *dcw* gene cluster have an uncommonly long 5’ UTR of 268 nt in length, which has not been reported for other bacterial *dcw* clusters, which show high conservation among rod shaped and gram negative bacteria. 2.) UpsM is not an sRNA derived from an IGR (intergenic region), but rather an sRNA which is generated by transcription termination within the 5’ UTR of *mraZ*. 3.) In case the terminator allows read-through, the 5’ UTR contains a start codon upstream and a stop codon in frame downstream of the terminator and therefore might encode a leader peptide here designated as sORF (small open reading frame).

### UpsM Is a 5’ UTR Derived sRNA, Which Is Conditionally Cleaved during Stress Conditions in an RNase E, Hfq and RpoHI/RpoHII-Dependent Manner

UpsM was originally identified as an sRNA processed upon ^1^O_2_ stress in *Rhodobacter sphaeroides* and the RNA chaperone Hfq was shown to be required for this processing [6]. UpsM is the most abundant sRNA in *R*. *sphaeroides* and represents about 60% of all Hfq bound sRNAs [12]. In addition to the strong interaction of UpsM with Hfq we observed a negative growth effect under anaerobic and aerobic conditions (S2B Fig) and changes in the transcriptome in an initial microarray analysis (S1 Table) for a strain overexpressing UpsM (S2A Fig). Therefore we conclude that UpsM is functional as a *trans* acting sRNA.

**Fig 2:**
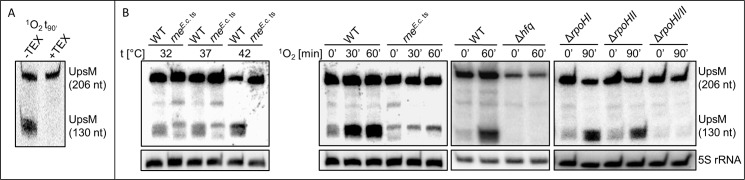
Northern blot analysis reveals Hfq and target mRNA dependent processing of UpsM by RNase E. (A) Detection of UpsM (206 nt) and the UpsM processing product (130 nt) by Northern blot analysis of TEX treated and untreated total RNA isolated from *R*. *sphaeroides* after 90 min ^1^O_2_ stress. (B) Left: Comparison of the UpsM processing pattern via Northern blot analysis of total RNA isolated from *R*. *sphaeroides* 2.4.1 (WT) to RNA isolated from a mutant strain expressing a thermosensitive RNase E variant from *E*. *coli* (*rne*^*E*.*c*.ts^) after growth at 32, 37 or 42°C for 30 min or during ^1^O_2_ stress. Right: Comparison of the processing pattern via Northern blot analysis to strains lacking Hfq, RpoHI, RpoHII or RpoHI and RpoHII after 0 and 60 or 0 and 90 min of ^1^O_2_ stress. Signals of 5S rRNA serve as loading control.

To further prove that the stable 130 nt UpsM 3’ fragment is generated by processing, total RNA from *R*. *sphaeroides* isolated 90 min after addition of methylene blue in the light to generate ^1^O_2_ was treated with TEX (terminator-5´phosphate dependent exonuclease), which degrades RNAs with a monophosphate at the 5´ end but not primary transcripts that carry a 5´ triphosphate. The TEX treated RNA was compared to untreated RNA on a Northern blot (Fig 2A). The lack of the 130 nt band after TEX treatment strongly supports the assumption that this fragment is a processing product.

In gram-negative bacteria the endoribonuclease RNase E has a major role in initiation of mRNA decay [13, 14] and was also shown to be involved in the generation or processing of several sRNAs [15–18]. To test the involvement of RNAse E in UpsM processing we constructed an *R*. *sphaeroides* strain with impaired RNase E activity. The endogenous *rne* gene (RSP_2131) was replaced by the *rne* gene from *E*. *coli* N3431 (46% blastp identity) [19, 20], which produces a temperature-sensitive RNase E due to a point mutation. As seen in Fig 2B processing of UpsM in strain *R*. *spharoides rne*^E.c.ts^ is already impaired at 32°C or 37°C, indicating that the *E*. *coli* enzyme is less active than the endogenous enzyme of *R*. *sphaeroides*. When cells are shifted to 42°C no UpsM processing occurs in the *R*. *sphaeroides* strain expressing the temperature-sensitive RNase E variant. When methylene blue was added to the cultures in the light growing at 32°C accumulation of the 130 nt UpsM fragment was only weak in the *rne* mutant strain, while a strong accumulation was observed in the wild type. As shown previously a lack of Hfq also abolished UpsM processing (Fig 2B) [6].

Deletion of the endonuclease RNase III or the 3’ to 5’ exonuclease RNase J does not lead to an altered processing (S3A Fig), showing that those nucleases are not involved. These results demonstrate that processing of UpsM is catalyzed by RNase E. This raises the question, why RNase E dependent processing occurs only under certain growth conditions. It is highly unlikely that all conditions leading to UpsM processing go along with increased RNase E levels, increased RNase E activity or structural changes in UpsM that promote cleavage by RNase E. It is known that sRNAs are often processed together with their target mRNA [15–18]. The Hfq protein can favor sRNA-mRNA interaction and the subsequent processing [15, 21]. Thus, it is conceivable that induction of the UpsM target(s) is responsible for appearance of the 130 nt processing product under the given conditions. To date (the) target mRNA(s) of UpsM are not identified. To better define the conditions, which promote UpsM processing, we tested the effect of further stress factors and growth conditions on the UpsM pattern (S3B and S3C Fig). Neither SDS, nor ethanol, hydrogen peroxide, tBOOH nor superoxide induced processing of the UpsM transcript (S3B Fig). High NaCl concentrations induced slight processing, while CdCl_2_ had a similar effect on processing as ^1^O_2_. Heat shock resulted in very fast UpsM processing (S3C Fig). The processing product was also clearly visible in RNA isolated from stationary phase cultures.

In *R*. *sphaeroides* the alternative sigma factors RpoHI and RpoHII stimulate many genes in response to stress conditions including ^1^O_2_, CdCl_2_, heat and stationary phase [22–26]. Northern blots revealed that normal UpsM processing in presence of ^1^O_2_ occurs in mutants either lacking RpoHI or RpoHII. However, a mutant lacking both sigma factors fails to process UpsM even under ^1^O_2_ stress (Fig 2B). We conclude that interaction with a target RNA very likely promotes UpsM cleavage by RNase E, whereas the target RNA is transcribed from a promoter, which is recognized by RpoHI as well as by RpoHII. It is known that the regulons of these two sigma factors indeed overlap [23]. No genes for known RNases are part of the RpoHI/RpoHII regulon [23, 24, 27] and UpsM heterologously expressed in *E*. *coli* does not show any induced processing even under stress conditions (data not shown) supporting the assumption of target-dependent processing.

### The *Dcw* Gene Cluster Features a Long 5’ UTR

We performed reporter assays, 5’ RACE (*rapid amplification of cDNA ends*) and RT-PCRs to address the question whether *mraZ* transcription is exclusively dependent on the UpsM promoter and whether the UpsM terminator allows read-throughs in order to guarantee transcription of *mraZ*. Conversely, this means that no additional promoter is localised between the UpsM promoter and *mraZ*.

**Fig 3:**
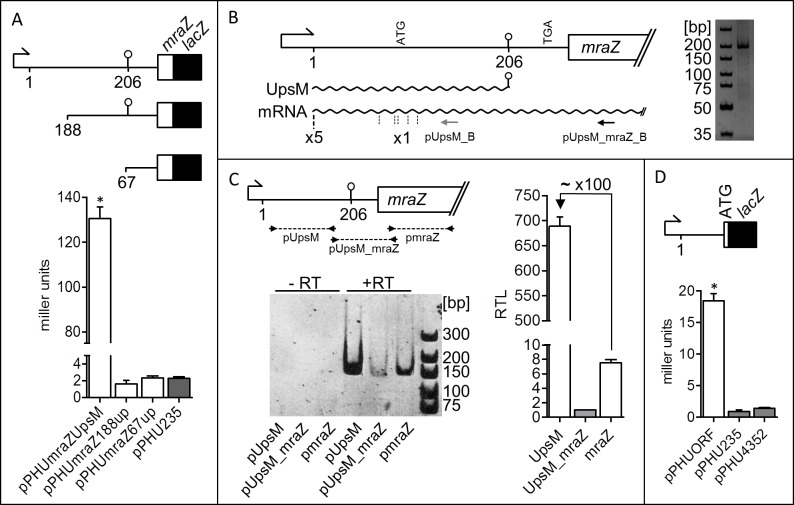
Transcription of *mraZ* is enabled by the UpsM promotor. (A) β-galactosidase activity assays of *R*. *sphaeroides* 2.4.1 with reporter plasmids with *mraZ*::*lacZ* translational fusion and *mraZ* upstream regions of varying length (long upstream region including the promotor of UpsM, 188 and 67 upstream nucleotides). pPHU235 represents the empty vector control. For each strain, three independent biological experiments with technical duplicates were performed. Error bars indicate standard deviations and an asterisk a significance level of P<0.01 compared to pPHU235. (B) 5’ RACE with RNA from *R*. *sphaeroides* 2.4.1 after 90 min of ^1^O_2_ stress. cDNA was generated with the primer depicted as black arrow, whereas cDNAs were amplified by the primer shown as grey arrow. The PCR product was visualized on a gel (10% PAA/TBE) by ethidium bromide staining. 5’ ends (dashed lines) identified by subcloning and sequencing and their corresponding frequencies are highlighted. (C) qRT-PCR products of primer pairs pUpsM, pmraZ (155 bp and 153 bp, both specific for the corresponding mRNA segments) and pUpsM_*mraZ* (143 bp, spanning from UpsM to *mraZ*) visualized on a gel (10% PAA/TBE) by ethidium bromide staining. Samples without initial RT step were loaded as control. On the right relative transcript levels are shown in relation to the product quantity of primer pair pUpsM_*mraZ*. qRT-PCRs were performed in technical duplicates with RNA from three biological independent and unstressed *R*. *sphaeroides* 2.4.1 cultures. Error bars indicate standard deviations. (D) β-galactosidase activity assays of *R*. *sphaeroides* 2.4.1 conjugated with a reporter plasmid with translational *lacZ* fusion to the start codon (ATG) within the UpsM gene in comparison to the promoter-less empty vector control (pPHU235) and a control plasmid (pPHU4352) containing a strong 16S rRNA promoter. For each strain, three independent biological experiments with technical duplicates were performed. Error bars indicate standard deviations and an asterisk a significance level of P<0.01 compared to both controls.

To test this we performed β-galactosidase activity assays by using reporter plasmids with *mraZ*::*lacZ* translational fusion and *mraZ* upstream regions of varying length in *R*. *sphaeroides* (Fig 3A). Strong activity of 130 Miller units was observed with plasmid pPHU*mraZ*UpsM containing the UpsM promoter. However, all shortened upstream regions of *mraZ* (188 nt or 67 nt) led to β-galactosidase activities similar to that observed for the empty vector control (pPHU235) proving the absence of any additional promoter closer to *mraZ*.

To further support this assumption we conducted a 5’ RACE to determine 5’ ends of *mraZ* mRNAs, whereby cDNA synthesis was enabled by a primer (pUpsM_mraZ_B) binding within the coding region of the *mraZ* gene. Further amplification of cDNA was done with a primer located upstream (pUpsM_B). The resulting PCR products are visible as one clear band on a gel and show the migration behaviour of fragments with 194 bp length which corresponds to the 5’end of UpsM (Fig 3B). The amplified DNA fragments were also subcloned into the pDrive vector without any further purification to determine the 5’ ends precisely. The 5’ end of UpsM was found in five of ten sequences, whereas the other 5’ ends were distributed randomly probably due to technical reasons (Fig 3B). This experiment confirms, 1) that *mraZ* transcription depends on the promoter of UpsM, 2) the terminator of UpsM allows read-throughs leading to *dcw* transcription and 3) the 5’ UTR of *mraZ* respectively *dcw* mRNAs is not processed like UpsM, since we used RNA from cells after 90 min ^1^O_2_ stress and did not detect the 5’ end of UpsM (130nt).

Furthermore we demonstrated read-throughs at the UpsM terminator and estimated the frequency of such events by RT-PCRs and an unconventional qRT-PCR approach with DNA free RNA from unstressed exponentially grown cultures. The two primer pairs pUpsM_A/B and pmraZ_A/B specifically amplify an UpsM or *mraZ* segment respectively (155 bp and 153 bp), whereas primer pair pUpsM_mraZ_A/B amplifies a segment (143 bp) spanning from UpsM to *mraZ* and therefore detects the read-through. RT-PCR products for all primer pairs are clearly visible on a gel, whereas none of those fragments emerge in control samples without prior reverse transcription (Fig 3C). For a rough estimate of the read-through frequency we compared amplification cycles or rather Cq values in qRT-PCR between all primer pairs in the same RNA samples instead of between different RNA samples with the very same primer pair. Since product sizes are very similar the fluorescent dye will intercalate comparably into *de novo* DNA. The mRNA level of the segment representing the read-through seems to have the lowest abundance. However, this is probably due to a terminator mediated bias and the smaller product size. In Fig 3C the relative transcript levels calculated in comparison to 16S rRNA levels are shown in relation to the transcript level detected by primer pair pUpsM_mraZ. Approximately 700 and 7 times higher RNA levels corresponding to UpsM and *mraZ* respectively were detected. As demonstrated *mraZ* transcripts have long 5’ UTRs and contain the UpsM locus. Therefore part of the DNA amplified by the primer pair pUpsM originates from full-length transcripts also encoding *mraZ*. In other words, *mraZ* transcripts are amplified not only by the primer pair pmraZ but also by pUpsM primers. By taking this into consideration we estimate that one read-through event or rather *mraZ* transcription takes place about once in 100 transcription events under the given experimental conditions.

We were able to show convincingly that *mraZ* has a long 5’ UTR under transcriptional control of the UpsM promoter. In addition the 5’ UTR contains a start codon upstream and a stop codon in frame downstream of the terminator of UpsM and therefore might encode a leader peptide here designated as sORF (small open reading frame). To test translation initiating at this start codon we performed β-galactosidase activity assays of a reporter plasmid with an sORF::*lacZ* translational fusion containing the upstream region including P_UpsM_ in *R*. *sphaeroides*. The sORF::*lacZ* fusion on plasmid pPHUORF resulted in low but significant higher β-galactosidase activities of approximately 18 Miller units in comparison to the promoter-less empty vector control pPHU235 and control plasmid pPHU4352 containing a strong 16S rRNA promoter (Fig 3D). This indicates translation of the ORF, but final proof by a direct detection of the hypothetical peptide is missing.

### The 5’ UTR of the *Dcw* Gene Cluster in *Rhodobacteraceae* Differs from Other Bacteria

The upstream region of the *mraZ* gene was compared to that of other species. Using public available deep sequencing data obtained from the NCBI SRA database [28], we annotated TSS (transcription start sites) within 300 nt upstream of the gene locus. Reads were mapped to the respective genomes using segemehl [29] with default parameters after quality trimming using Trimmomatic [30] at a quality threshold of 25 in a sliding window of size 3. Only reads of size >14 nt were considered. See S4 Fig for details. Rho-independent Terminators in the 5’-region were predicted using TransTermHP [31]. The highest scoring hit was assumed to be the terminator whenever its MFE (minimum free energy) was below -10 kcal/mol according to RNAfold [32].

**Fig 4:**
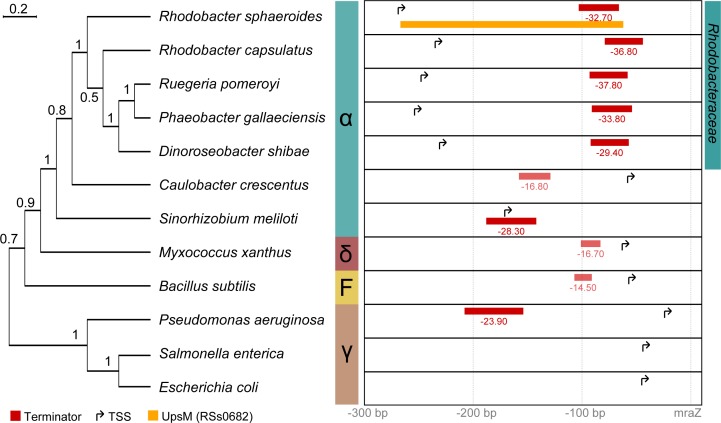
Comprehensive view of *mraZ* upstream regions in different species. Terminator predictions are indicated in red. Respective energies are given in kcal/mol. Regions between Start and Stop codons in frame are shown as grey bars. Transcription start sites are derived from public available deep sequencing data (see S4 Fig for details). The phylogenetic tree was build using clustalx [58] (NJ, 10000 bootstraps) based on a clustalOmega [59] alignment of the respective *mraZ* coding regions. Bootstrap support values are indicated. Seemingly the long *mraZ* 5’ UTR with an intrinsic terminator is special to the family of *Rhodobacteraceae*.

A summary is shown in Fig 4. *Rhodobacteraceae* consistently show a long 5’ UTR with a strong terminator and no additional TSS close to *mraZ* in a particularly striking manner. In contrast, other species apart from *Rhodobacteraceae* typically have an exclusive TSS closer to *mraZ* resulting in 5’ URTs of ≤ 70 nt, including the closely related Alphaproteobacterium *Caulobacter crescentus*. Moreover upstream terminators are not found at all or are predicted to be rather weak in those species. A notable exception is *Sinorhizobium meliloti* by exhibiting 5’ UTR mediocre in length (170 nt) whereby the TSS is located within a predicted terminator site. However, whether this upstream TSS contributes to the *mraZ* expression as shown for *R*. *sphaeroides* remains speculative but has never been reported. Taken together our data suggests that a long *mraZ* 5’ UTR with intrinsic terminator generating an sRNA combined with no separate TSS for *mraZ* is an exclusive feature of the family of *Rhodobacteraceae*.

### Predicted Secondary Structure and Folding Landscape of UpsM

**Fig 5:**
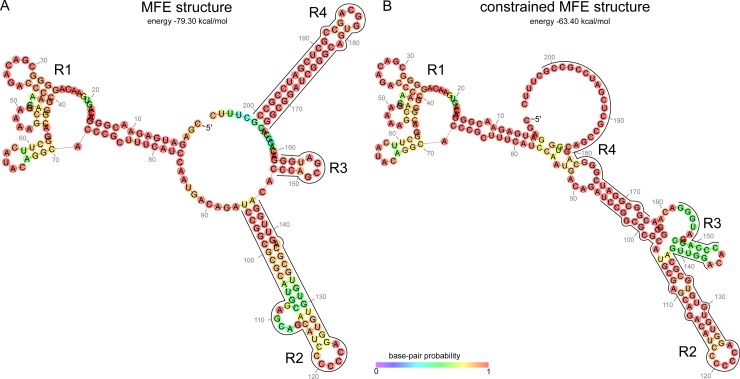
Structural analysis of UpsM. Analogous structured regions are indicated as R1-R4. RNAfold structure of UpsM in *R*. *sphaeroides* with and without constraint terminator (R4).

The secondary structure prediction of UpsM was evaluated *in silico* using RNAfold [32]. It consists of four structured regions (R1-4). R1 is located at the 5’ end. It consists of several short hairpins isolated from the remaining RNA by a ~11 nt long stem followed by an unpaired region of 10 nt. R2 is a long hairpin with a small bulge, R3 a short hairpin with a stem of only 4 nt. R4 is located at the 3’ end of the molecule and corresponds to the predicted, strong terminator structure (Fig 5A).

The secondary structure elements R2/3 and R4 show similarities to typical riboswitches (R2/3 aptamer region, R4 terminator) [33]. Hence we constrained the fold such that the terminator would not form. Strikingly, the refolding event leads to an interaction of R2 and R4 owing a loss of ~20% energy (15.9 kcal/mol) (Fig 5B). Based on this analysis, the *dcw* 5’ UTR has the potential to represent a riboswitch.

To verify our assumption, we aligned the 5’-UTR regions of all *Rhodobacteraceae* in our dataset (cropped at the predicted terminator) using mlocarna [34] which respects sequence and secondary structure at the same time. The alignment was folded using RNAalifold from the Vienna RNA 2.0 package [32] with no constraints and, analogously to above, constraint such that the terminator would not be allowed to form. S5A Fig shows the resulting structures with conservation. S5B Fig indicates the underlying alignment with secondary structure indications.

The consensus structure implies that R1 is hardly conserved. Moreover, the predicted transcription start site is further downstream in other species, leading to shorter transcripts (e.g. 19 nt of R1 are missing in *D*. *shibae*). We thus assume that R1 is relevant for the function of UpsM. Other than that, the consensus structure well resembles the UpsM structure, indicating that R2-4 are conserved among *Rhodobacteraceae*. Constraint folding however does not induce an interaction of R2 and R4 in this case. Hence, the putative riboswitch function is seemingly not conserved in *Rhodobacteraceae* but, if so, an evolutionary trait special to *R*. *sphaeroides*.

The folding landscape of UpsM was predicted using RNAsubopt, RNAfold and barriers from the Vienna RNA 2.0 package [32] as well as treekin [35]. A summary is shown in S6 Fig. The four most probable structures of UpsM mainly differ in the terminator hairpin (R4, states 1, 4 and 12) while one variant slightly differs in R1. States 1 and 4 represent the most pronounced folding minima with the highest probability (S6A and S6B Fig). Potential interactions between the structured regions are not observed. These findings indicate a rather stable structure that requires an additional partner (e.g RNA, protein or ligand) for substantial refold.

## Discussion

In this study we further characterize the sRNA UpsM (previously RSs0682) and demonstrate that it is derived from the 5´ UTR of the mRNA for *mraZ*, the first gene of the *dcw* gene cluster. Overexpression of UpsM leads to a mild growth defect and to a change in the global gene expression pattern as well under aerobic conditions as under photooxidative stress conditions, proving that UpsM is also functional in *trans*. Further experiments need to clarify whether altered mRNA levels are due to direct base pairing or rather to binding of high amounts of Hfq. Analysis of a dRNA-seq dataset of high coverage and Northern blot analysis of TEX treated RNA from ^1^O_2_ stressed cultures confirmed a processing step from UpsM (206nt) to UpsM (130nt). The UpsM processing pattern under different stress conditions and in various mutant strains showed that processing requires the RNA chaperon Hfq, the endoribonuclease RNase E and the alternative sigma factors RpoHI/II. mRNAs being part of the RpoHI/II controlled stress regulon are induced similar to the processing of UpsM upon ^1^O_2_ stress [6, 12, 23], heat stress [24] and in stationary phase (unpublished). Therefore we assume that UpsM is bound and stabilized by Hfq prior to target recognition. However, under stress conditions a target mRNA is expressed which may form a duplex with UpsM mediated by Hfq. In the course of base pairing the structure of UpsM might be altered and becomes susceptible to RNase E cleavage. A similar mechanism was first described for the Hfq dependent sRNA RyhB, which is degraded in an RNase E dependent manner upon binding to the target mRNA *sodB* in *E*. *coli* [15]. Moreover, an interaction of Hfq with the scaffolding domain of RNase E for the purpose of a recruitment of RNase E to sRNA-mRNA hybrids has been discussed [36] and might also be true for *R*. *sphaeroides*.

The dRNA-seq data of high coverage based on exponentially growing *R*. *sphaeroides* cultures, did not show a TSS exclusive for *mraZ*, despite the fact that *mraZ* represents the first gene of the *dcw* gene cluster. Therefore and based on dRNA-seq data we hypothesized that transcription of *mraZ* depends on the UpsM promoter, which implicates that the terminator of UpsM allows read-throughs in order to guarantee transcription of *mraZ* with a long 5’ UTR of 268 nt in length. We were able to proof this assumption by 5’ RACE and reporter assays with different fragments of the *mraZ* 5´ UTR and estimated the read-through at the UpsM terminator to takes place once in 100 transcription events under our experimental conditions. This might ensure sufficient proximal *dcw* gene transcription, since UpsM is the most frequently transcribed sRNA in *R*. *sphaeroides* [6]. Taken together our data demonstrates that *mraZ* or polycistronic *dcw* gene mRNAs of *R*. *sphaeroides* feature long 5’ UTRs, with the consequence that UpsM has to be reckoned as a 5’ UTR derived sRNA rather than an orphan sRNA derived from an IGR as previously assumed [6].

In recent years transcriptome wide identification of sRNAs revealed that apart from intergenic regions especially 3’ UTRs serve as a reservoir for sRNAs being part in the Hfq network [37, 38] and their functions in *trans* was reported [39–41]. In comparison, 5’ UTR- or riboswitch-derived sRNAs have been described only occasionally and in general without clear functional assignment in *trans*. A handful of putative sRNAs resulting from processed 5’ UTRs are mentioned in a transcriptome study of *Yersinia pseudotuberculosis* [42]. For *E*. *coli* prematurely terminated transcripts from 5’ leader sequences of *ybjM*, *ynaE*, *ydfK*, *mdtJ*, *typA*, *yhiI*, and *dinQ* were detected [43], among which *ynaE*, *ydfK* and *ybjM* 5’ leader fragments co-immunoprecipitate with Hfq [44]. In the same study an sRNA corresponding to a 5’ UTR segment of the *adhE* mRNA was identified, possibly generated by RNase III [44]. A cloning based screen for ncRNAs in *E*. *coli* led to the discovery of a few 5’ UTR derived RNA fragments, which correspond to riboswitches or to be precise L-box, THI box and RFN box elements known to be required for the regulation of mRNA or protein synthesis by attenuation or RBS accessibility [45]. However, neither an association to Hfq nor any other proof for a function of those RNA fragments in *trans* was provided in this study. Recently 14 sRNAs which also might serve as 5’ UTRs were predicted in *Vibrio cholera*, but only for Vcr043 an Hfq dependent stability was shown indicating a function in *trans* [46]. To our knowledge and surprisingly only once dual function 5’ UTRs were described so far, which act as riboswitch and generate sRNAs in the course of attenuation with regulatory function in *trans*. This was described for SreA and SreB, two S-adenosylmethionine (SAM) riboswitches in *Listeria monocytogenes*. SreA and SreB are transcribed together with downstream genes encoding proteins involved in methionine and cysteine transport or metabolism. Upon binding of the ligand SAM transcription is attenuated causing expression of SreA and SreB as short transcripts, which control expression of the virulence regulator PrfA precisely as sRNAs by binding to the 5‘ region of its mRNA [47]. The authors assumed SreA/SreB to be just the first example of a novel distinct class of riboswitch derived sRNAs, without expecting that no further example was reported over the past seven years. In this study we were able to describe a similar example, since UpsM can be transcribed with the downstream gene, but is also generated as stable sRNA by an intrinsic terminator in the 5’ leader. However, we were only able to provide evidence for a function of UpsM in *trans* by strong Hfq dependency and target dependent cleavage of the sRNA, whereas we cannot present any experimental evidence for a riboswitch at the intrinsic terminator in the leader sequence so far. Its prominent position in the 5’ UTR of the *dcw* gene cluster and combined with the potential breakup the terminator structure R4 due to an interaction with R2, however, makes UpsM a reasonable candidate for a riboswitch. Regulatory features and a potential function as riboswitch will be subject to future investigation.

The regulatory features of the *mraZ/dcw* 5’ UTR might be even more complex, since we have indications for weak translational activity at an sORF overlapping with the terminator of UpsM. *Cis*-regulatory elements as such sORFs sometimes also termed leader peptides or uORFs (upstream open reading frame) in eukaryotes are often involved in transcriptional or translation attenuation [48, 49]. However, this sORF is only present in *R*. *sphaeroides* species and presence of the resulting peptide is not unequivocally proven.

The *mraZ/dcw* 5’ UTR or rather UpsM may represent not only the second example for a class of riboswitch derived sRNAs, but also is the first extended 5’ UTR described for the *dcw* gene cluster, despite outstanding conservation among rod shaped and gram negative *bacteria* [1–3]. In *E*. *coli* the first promoter (*mraZ*1*p*) of the gene cluster (16 genes in total) gives rise to polycistronic transcripts containing a short 38nt long 5’ UTR followed by the first gene *mraZ* [4, 7]. However, in this study we have been able to describe a much longer 5’ UTR of 268 nt in length featuring *trans-*regulatory and potential *cis*-regulatory elements (summarized in S7 Fig). Therefore we were interested whether we would find similar 5’ leaders in the same genetic context of other bacteria. Our results suggest that long 5’UTRs with intrinsic terminators are exclusively present in members of *Rhodobacteraceae*.

## Material and Methods

### Bacterial Strains and Growth Conditions

Bacterial strains used in this study are listed in S2 Table. Details on their construction are given in S1 File. *R*. *sphaeroides* strains were cultivated at 32°C in malate minimal-salt medium [50]. To grow the cells aerobically, cultures were either gassed with air in Meplat bottles to attain a concentration of 160 to 180 μM of dissolved oxygen or by continuous shaking of Erlenmeyer flasks containing 20% culture by volume at 140 rpm. For microaerobic growth conditions, having a dissolved oxygen concentration of about 25 μM, Erlenmeyer flasks containing 80% culture by volume were shaken at 140 rpm. For anaerobic growth in the dark in the presence of 60 mM DMSO as electron acceptor we used completely filled screw-cap Meplat bottles, completely filled and sealed with Parafilm. When necessary tetracycline (2 μg ml^-1^), kanamycin (25 μg ml^-1^) or spectinomycin (10 μg ml^-1^) was added to liquid and solid growth media (1.6% agar). Photooxidative stress conditions were generated as described earlier [51], except the final concentration of methylene blue (0.2 μM) (Sigma-Aldrich; M9140). Other stress conditions were generated by a final concentration of 250 mM NaCl, 10 μM CdCl_2_, 0.005% SDS, 2.5% ethanol, 300 μM tBOOH, 1 mM H_2_O_2_ and 250 μM paraquat (O_2_^-^) or by temperature shift to 42°C. To culture *E*. *coli* strains, cells were continuously shaken at 180 rpm in Luria–Bertani medium at 37°C or grown on solid growth media containing 1.6% (w/v) agar. When necessary kanamycin (25 μg ml^-1^) or tetracycline (20 μg ml^-1^), ampicillin (200 μg ml^-1^) or spectinomycin (10 μg ml^-1^) was added to the media.

### Northern Blot Analysis

Northern blots were performed as described earlier [6]. Oligodeoxynucleotides used for end-labeling with [γ-^32^P]-ATP (Hartmann Analytic; SRP-301) by T4 polynucleotide kinase (Fermentas; #EK0031) are listed in S3 Table. A low stringency Church buffer was used for hybridization [52]. Membranes were washed in 5x SCC buffer + 0.1% SDS. After exposure on phosphoimaging screens (Bio-Rad), images were analyzed by the 1D-Quantity One software (Bio-Rad).

### Isolation of Total RNA

Total RNA used for Northern blot, 5’ RACE and real time RT-PCR was isolated by the hot phenol method [53]. To remove remaining traces of DNA, samples were treated with 6 U of DNaseI (Invitrogen; #18047019) per 1 μg of RNA. Absence of DNA contamination was confirmed by PCR with primers targeting *gloB* (RSP_0799) (S3 Table).

### 5’ RACE

To determine 5’ mRNA ends of *mraZ* using 5’ rapid amplification of cDNA ends (RACE), 3 μg of DNA free total RNA isolated from wild type cells after 90 minutes of ^1^O_2_ stress were reverse transcribed into cDNA by using avian myeloblastosis virus reverse transcriptase (Promega) and gene-specific primer pUpsM_mraZ_B (S3 Table). A second amplification was done with primer pUpsM_B (S3 Table). The 5′RACE protocol was performed as described previously [24].

### qRT-PCR

The One-Step Brilliant III QRT-PCR Master Mix Kit (Agilent) was used for reverse transcription and following PCR as described in the manufacturer’s manual but in 10 μl volumes containing 2 μl DNA free RNA in the concentration 0.2 ng/μl. Runs in independent biological triplicates with technical duplicates were done by the use of a Bio-Rad CFX96 Real Time System. Cq values at the auto calculated RFU were extracted with the corresponding software Bio-Rad CFX Manager. mRNA levels were calculated in relation to the mRNA levels of 16S rRNA similar to Pfaffl [54], but with fixed primer efficiencies of 2.0 and a fixed denominator of 1.0, since Cq values of different primer pairs were compared and the very same RNA sample was used. Therefore the resulting formula is: Ratio = 2^ΔCq(16S –A)^, whereas A is Cq of primer pair pUpsM, pUpsM_marZ or pmraZ. Primers are listed in S3 Table.

### β-Galactosidase Activity Assay

β-galactosidase activity was measured in conjugants obtained after transferring the respective reporter plasmids via di-parental conjugation from *E*. *coli* to *R*. *sphaeroides*. Three independent liquid cultures, inoculated with equal numbers of colonies, were grown microaerobically and diluted to OD_660_ 0.2, before reaching stationary phase. Three samples of 1 ml were collected in early exponential growth phase (OD_660_ 0.4). Measurements of β-galactosidase activity were carried out as described previously [22].

### RNA Treatment, Library Preparation and Sequencing

DNA free total RNA isolated from exponential and microaerobic cultures were treated with TEX (Epicentre #TER51020) to enrich primary transcripts [55]. For *R*. *sphaeroides* RNA Illumina cDNA libraries were prepared by vertis Biotechnology AG, Germany (http://www.vertis-biotech.com/) as described before without prior RNA fragmentation or size fractionation [50]. Illumina cDNA libraries resulting from TEX treated *R*. *capsulatus* RNA were generated as described before without prior rRNA depletion [42]. cDNA libraries were sequenced on a HiSeq 2000 or HiSeq 2500 machine in single-read mode running 100 cycles. Raw files for *R*. *sphaeroides* have been deposited in the National Center for Biotechnology Information Gene Expression Omnibus (GEO) [56] and are accessible via the GEO accession GSE71844. Raw files for *R*. *capsulatus* are accessible via BioProject Accession PRJNA343088.

### Microarray Analysis

Microarray analysis was performed as described before [26, 57]. Total RNA, obtained from 6 independent cultures per strain later hybridized with a duplicate of arrays was chemically labeled with Cy3 and Cy5 (Kreatech; EA-022/EA-023), respectively. Multiarray analysis was performed with the Bio-conductor package Limma for R. On the basis of calculated MA plots, genes were considered reliable if the average signal intensity [A-value: 1/2 log2 (Cy3×Cy5)] was ≥ 12. To filter out potentially insignificant changes among genes that passed the reliability criterion, a cutoff value was applied; i.e., those genes were retained whose average expression value of the overexpression strain (a) compared with the average value of the control treatment (b) was either a log_2_ fold change of ≥ 0.65 or ≤ -0.65. Microarray data are deposited in the Gene Expression Omnibus (GSE87789).

## Supporting Information

S1 Fig Nucleotide sequence of the *mraZ* 5’ UTR. Sequence of UpsM is shaded in grey. The corresponding terminator is underlined. The hypothetical sORF coding region starting with ATG and the *mraZ* coding region starting with GTG are depicted by bold letters.
(TIF)

S2 Fig (A) Altered UpsM transcript level shown by Northern blot analysis of total RNA of the overexpression strain *R*. *sphaeroides* 2.4.1 pBBRUpsMx2 after 60 min ^1^O_2_ stress in comparison to the wild-type strain harboring the empty vector (pBBR1MCS2). Signals of 5S rRNA serve as loading control. (B) Aerobic, microaerobic and anerobic growth of the overexpression strain *R*. *sphaeroides* 2.4.1 pBBRUpsMx2 in comparison to the wild-type strain harboring the empty vector (pBBR1MCS2). The optical density at 660 nm (OD_660_) was determined over time, and growth is indicated as continuous line. All graphs represent the mean of three biological independent experiments. Error bars indicate the standard deviation at each time point measured.
(TIF)

S3 Fig Processing pattern of UpsM under various ongoing stress conditions and in strains lacking RNases shown by Northern blot analysis of total RNA isolated from *R*. *sphaeroides*. (A) Processing pattern of UpsM in strains lacking RNase III (Δ*rnc*) or RNase J (Δ*rnj*). Signals of 5S rRNA serve as loading control. (B) Stress conditions were generated by a final concentrations of 0.005% SDS, 2.5% ethanol, 300 μM tBOOH, 1mM H_2_O_2_ and 250 μM paraquat (O_2_^-^). (C) Stress conditions were generated by a final concentrations of 0.2 μM methylene blue in the presence of 800 Wm^-2^ white light (^1^O_2_), 250 mM NaCl and 10 μM CdCl_2_ or by stationary phase or growth under heat stress at 42°C.
(TIF)

S4 Fig Genomic regions upstream of *mraZ* in other species. Underlying deep sequencing data was used to predict transcriptional start sites. Layout resembles Fig 4. Genome IDs are indicated at the y-axis. Sources are indicated at the right.
(TIF)

S5 Fig Structural analysis of UpsM. Analogous structured regions are indicated as R1-R4. (A) RNAfold structure of UpsM in *R*. *sphaeroides* with and without constraint terminator (R4) and consensus structure of aligned sequences for all *Rhodobacteraceae* without constraint terminator (R4). (B) RNAalifold alignment with structural annotation and indicated terminator constraint (x = bases forced to be unpaired). An interaction of R2 and R4 occurs when applying terminator constraints. This is however not resembled by the consensus structure.
(TIF)

S6 Fig Folding state analysis of UpsM. (a) Barrier tree of all suboptimal structures of the unprocessed sRNA. Four main species with favorable energies were identified (1, 4, 8, 12). (B) Population density of these structures over time (no unit). (C) Structural representations of the four most favorable states.
(TIF)

S7 Fig Model summarizing the results of this publication. The 268 nt long 5’ UTR of *mraZ*, first gene of the *dcw* (division and cell wall) gene cluster, comprises a Rho independent terminator, which in case of termination gives rise to the 206 nt long non-coding RNA UpsM (upstream sRNA *m**raZ*). Under stress conditions this sRNA is conditionally cleaved by RNase E in an Hfq- and likely in a target mRNA-dependent manner, whereas the corresponding target mRNA is controlled by an RpHI/II dependent promotor.
(TIF)

S1 File Strain construction. (PDF)

S1 Table Gene expression of an UpsM overexpression strain *R*. *sphaeroides* 2.4.1 pBBRUpsMx2 was analysed in comparison to the strain *R*. *sphaeroides* 2.4.1 pBBR1MCS2 harbouring the empty vector to get first insights into the biological function of UpsM. The transcriptome of both strains was compared by microarray analysis during exponential growth under aerobic and non-stress conditions and after 90 min of ^1^O_2_ stress. For both conditions a biological duplicate of arrays was hybridized with RNA from three biological independent cultures per strain. A Pearson correlation coefficient between the replica of 0.97 and 0.95 was calculated. Changes in expression levels of protein-coding genes passing the selection criteria of microarray analysis, which is a reliable A-value ≥ 12 and a log2 fold change of > 0.65 or < -0.65 between the two strains, are shown.
(DOCX)

S2 Table Strains and plasmids used in this study (DOCX)

S3 Table Oligonucleotides used in this study. (DOCX)
