# Supplementary figures and images for: The Conserved Dcw Gene Cluster of R. sphaeroides Is Preceded by an Uncommonly Extended 5’ Leader Featuring the sRNA UpsM
Source: PLoS One. 2016 Nov 1;11(11):e0165694. doi: 10.1371/journal.pone.0165694 (PMC5089854; doi:10.1371/journal.pone.0165694)

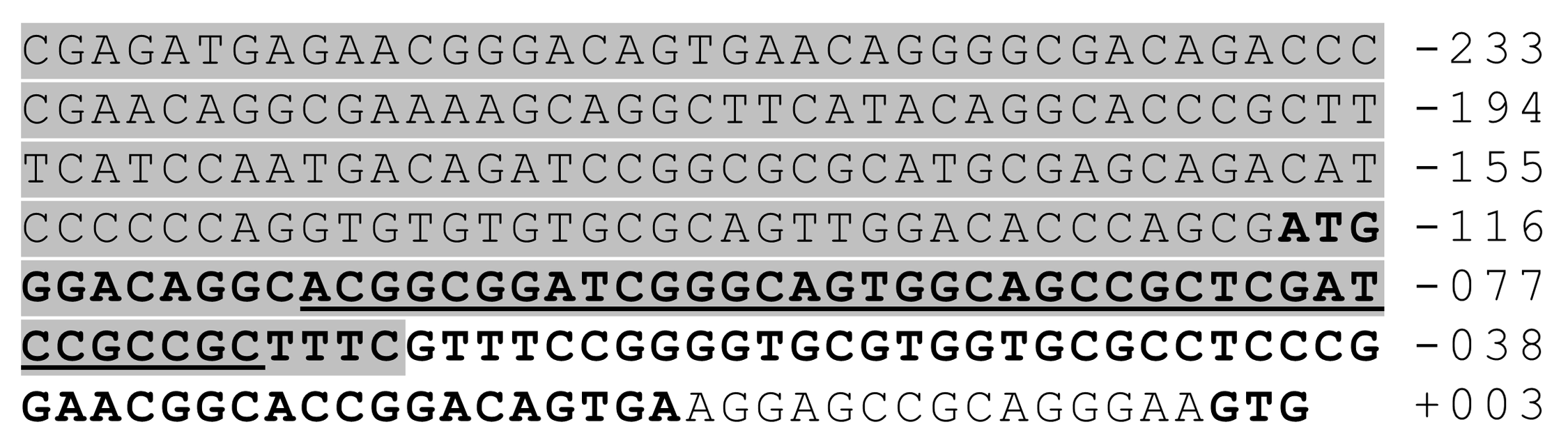

Supplement: S1 Fig — Sequence of UpsM is shaded in grey. The corresponding terminator is underlined. The hypothetical sORF coding region starting with ATG and the mraZ coding region starting with GTG are depicted by bold letters. (TIF) [file pone.0165694.s001.tif]

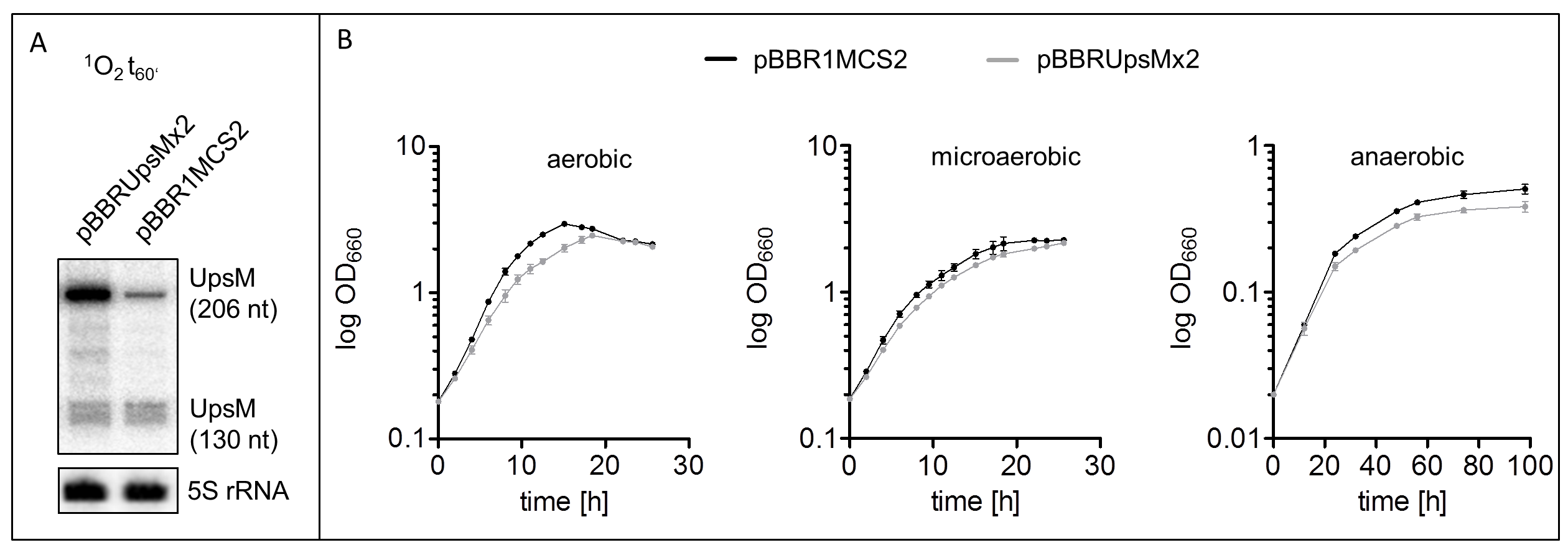

Supplement: S2 Fig — (A) Altered UpsM transcript level shown by Northern blot analysis of total RNA of the overexpression strain R. sphaeroides 2.4.1 pBBRUpsMx2 after 60 min 1O2 stress in comparison to the wild-type strain harboring the empty vector (pBBR1MCS2). Signals of 5S rRNA serve as loading control. (B) Aerobic, microaerobic and anerobic growth of the overexpression strain R. sphaeroides 2.4.1 pBBRUpsMx2 in comparison to the wild-type strain harboring the empty vector (pBBR1MCS2). The optical density at 660 nm (OD660) was determined over time, and growth is indicated as continuous line. All graphs represent the mean of three biological independent experiments. Error bars indicate the standard deviation at each time point measured. (TIF) [file pone.0165694.s002.tif]

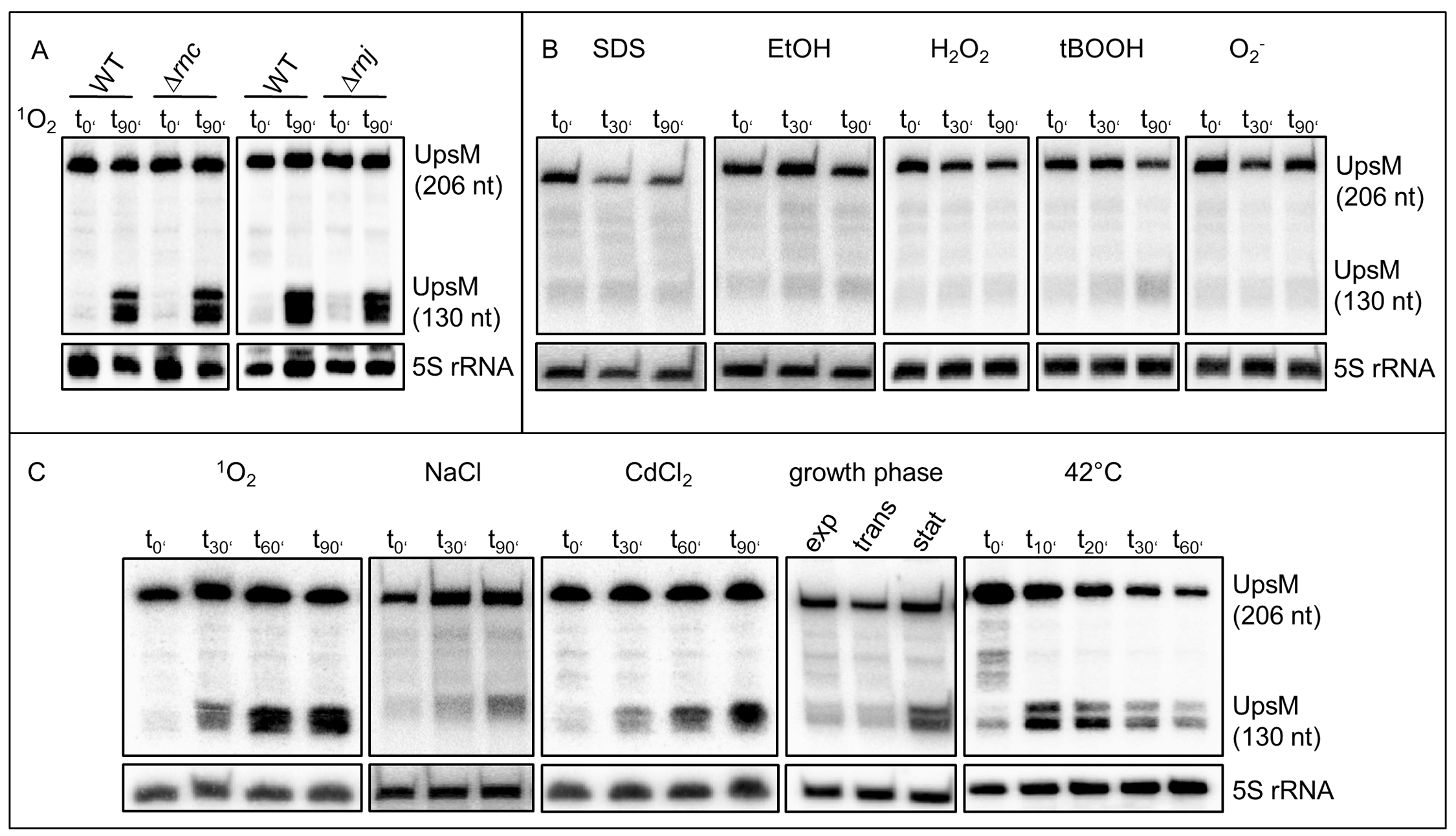

Supplement: S3 Fig — (A) Processing pattern of UpsM in strains lacking RNase III (Δrnc) or RNase J (Δrnj). Signals of 5S rRNA serve as loading control. (B) Stress conditions were generated by a final concentrations of 0.005% SDS, 2.5% ethanol, 300 μM tBOOH, 1mM H2O2 and 250 μM paraquat (O2-). (C) Stress conditions were generated by a final concentrations of 0.2 μM methylene blue in the presence of 800 Wm-2 white light (1O2), 250 mM NaCl and 10 μM CdCl2 or by stationary phase or growth under heat stress at 42°C. (TIF) [file pone.0165694.s003.tif]

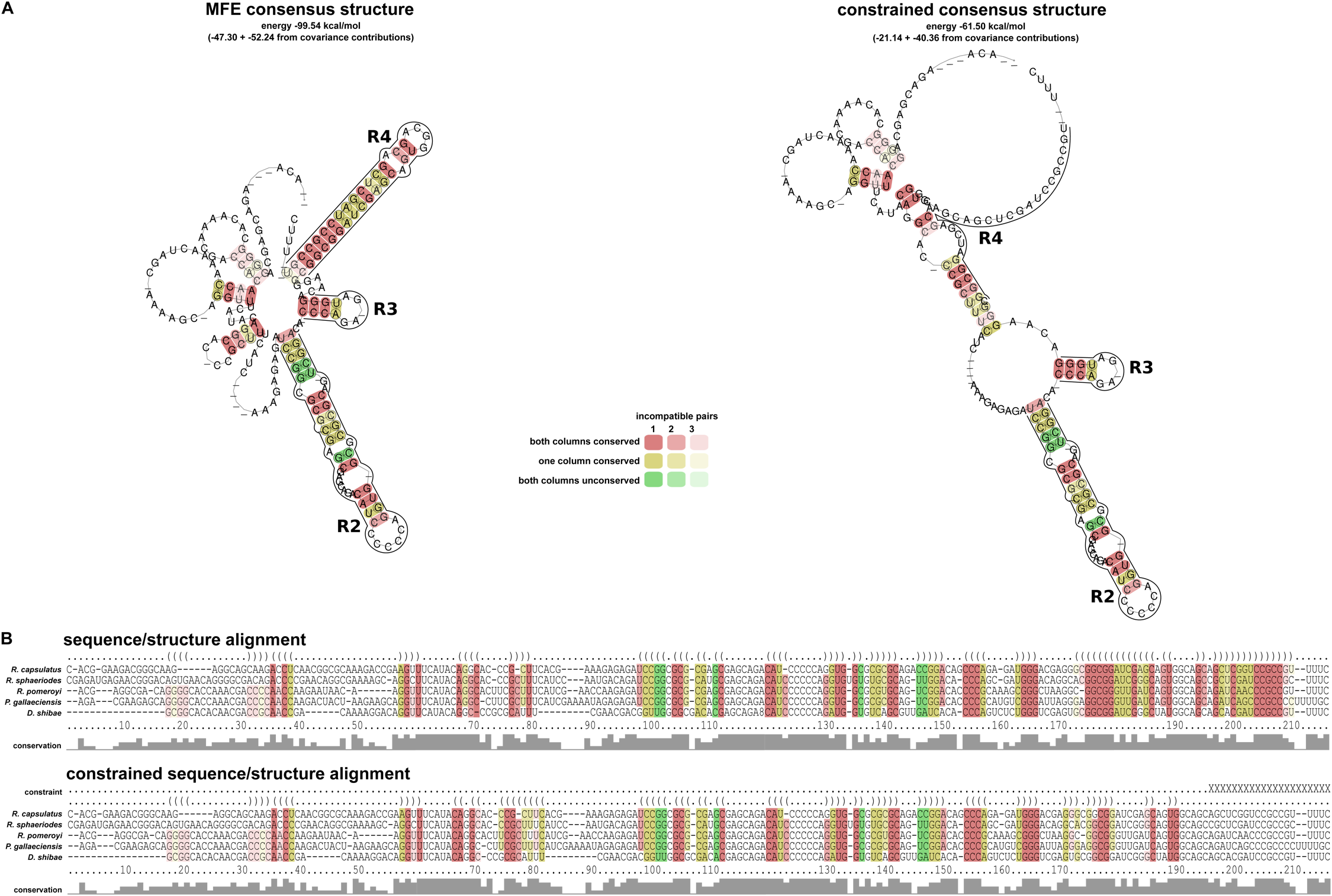

Supplement: S5 Fig — Analogous structured regions are indicated as R1-R4. (A) RNAfold structure of UpsM in R. sphaeroides with and without constraint terminator (R4) and consensus structure of aligned sequences for all Rhodobacteraceae without constraint terminator (R4). (B) RNAalifold alignment with structural annotation and indicated terminator constraint (x = bases forced to be unpaired). An interaction of R2 and R4 occurs when applying terminator constraints. This is however not resembled by the consensus structure. (TIF) [file pone.0165694.s005.tif]

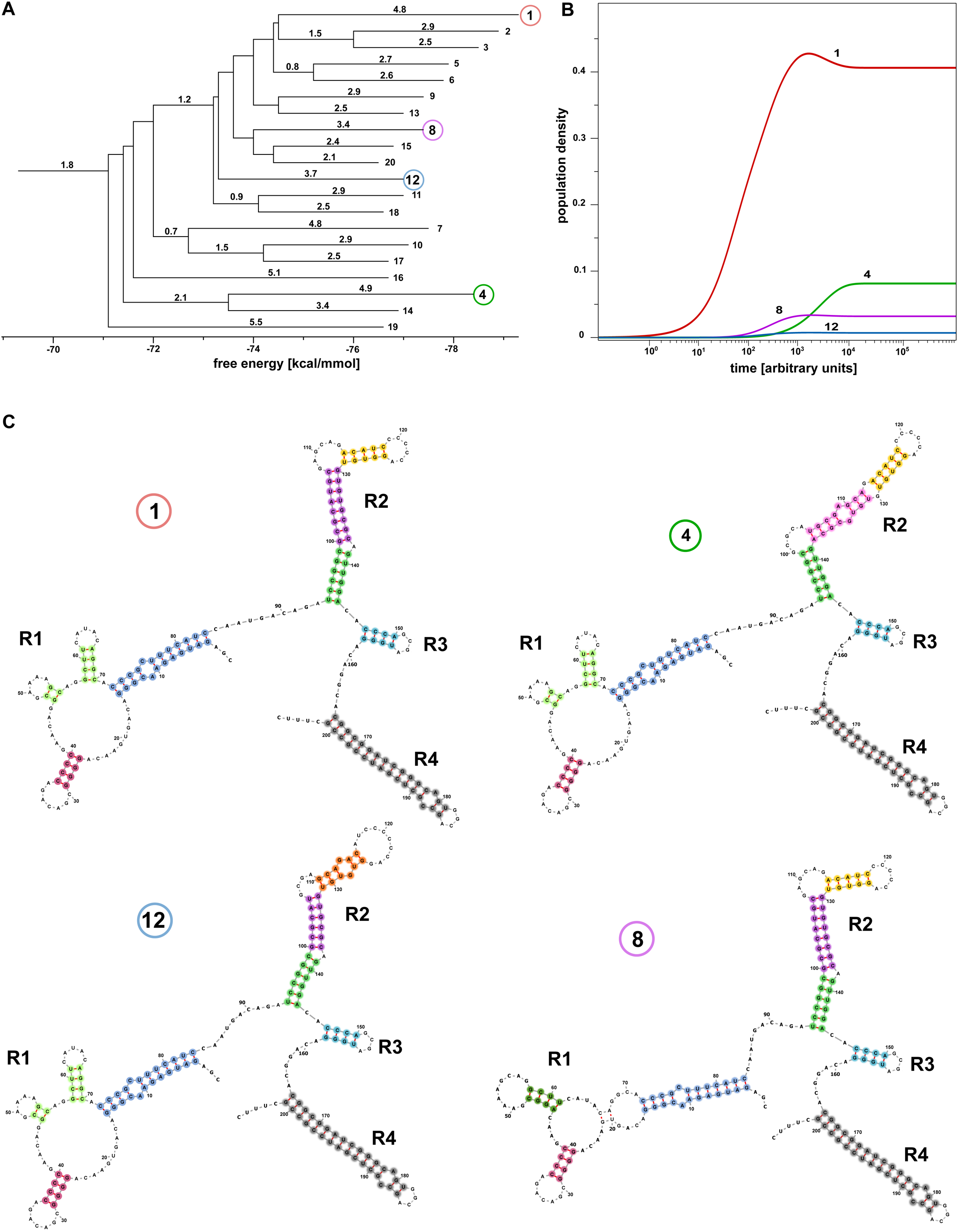

Supplement: S6 Fig — (a) Barrier tree of all suboptimal structures of the unprocessed sRNA. Four main species with favorable energies were identified (1, 4, 8, 12). (B) Population density of these structures over time (no unit). (C) Structural representations of the four most favorable states. (TIF) [file pone.0165694.s006.tif]

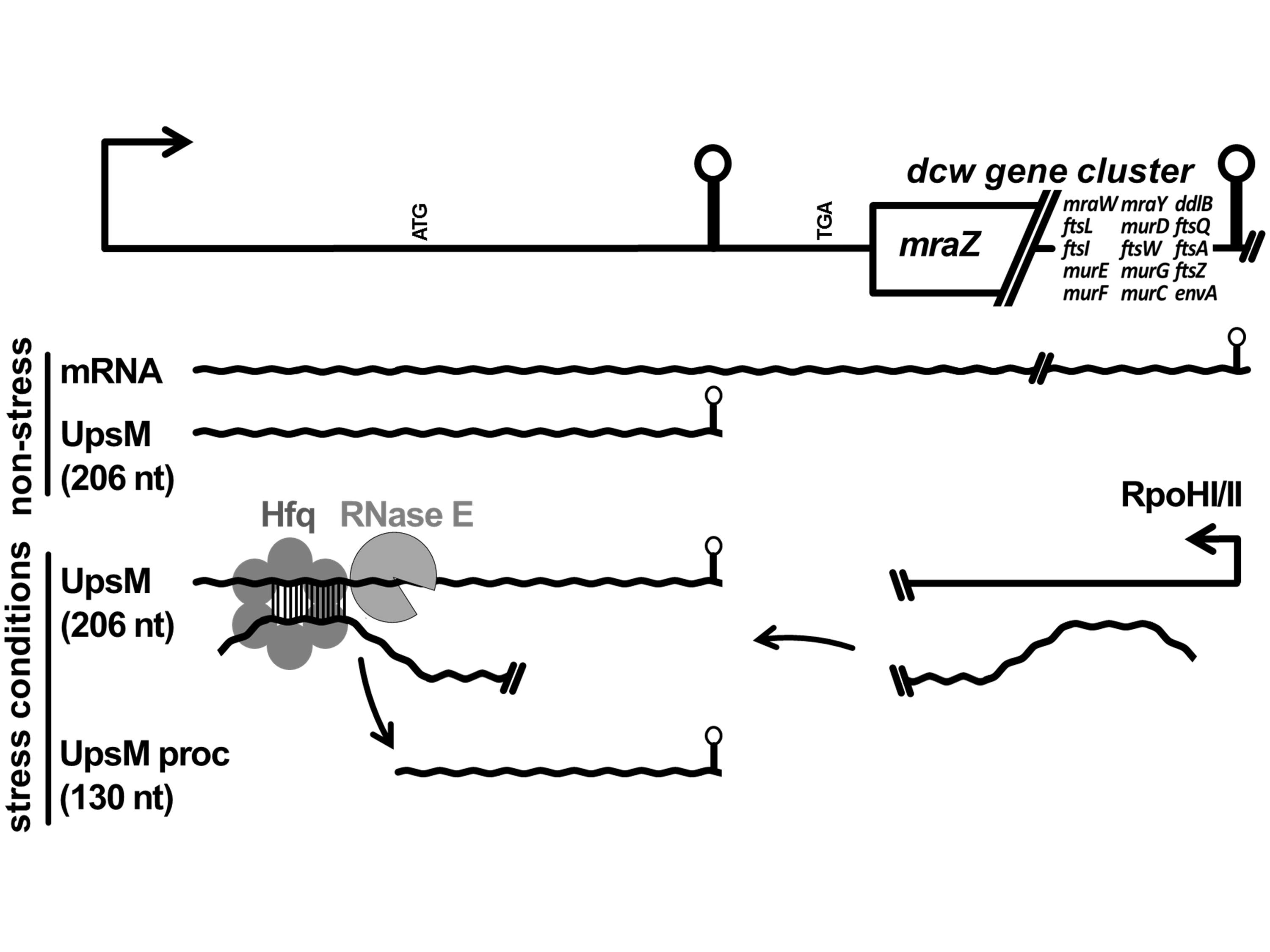

Supplement: S7 Fig — The 268 nt long 5’ UTR of mraZ, first gene of the dcw (division and cell wall) gene cluster, comprises a Rho independent terminator, which in case of termination gives rise to the 206 nt long non-coding RNA UpsM (upstream sRNA mraZ). Under stress conditions this sRNA is conditionally cleaved by RNase E in an Hfq- and likely in a target mRNA-dependent manner, whereas the corresponding target mRNA is controlled by an RpHI/II dependent promotor. (TIF) [file pone.0165694.s007.tif]
