# Supplementary material for: The Conserved Dcw Gene Cluster of R. sphaeroides Is Preceded by an Uncommonly Extended 5’ Leader Featuring the sRNA UpsM
Source: PLoS One. 2016 Nov 1;11(11):e0165694. doi: 10.1371/journal.pone.0165694 (PMC5089854; doi:10.1371/journal.pone.0165694)
Supplement: S1 File — (PDF) [file pone.0165694.s008.pdf]

S1 File. Strain construction.

Construction of strain *R. sphaeroides* 2.4.1 rne$^{\textit{E.coli}\textbf{(ts)}}$. This mutant encodes an RNase E variant with rne-*3071* (*ts*) mutation of *E. coli* N3431 [1, 2] instead of its native RNase E. Substitution of the gene locus was achieved by the suicide vector pPHU281_up_*rne*(ts)_sp/sm_down. For the construction upstream and downstream regions of *rne*$^{R.s.}$ for homologous recombination were amplified by PCR using the oligonucleotides R.s.rneUP_f, R.s.rneUP_r, R.s.rneDOWN_f and R.s.rneDOWN_r. *rne*$^{E.c.\mathrm{ts}}$ was amplified with oligonucleotides E.c.rne_fw and E.c.rne_rev and chromosomal DNA of strain *E. coli* N3431. R.s.rneUP_r and E.c.rne_fw generate NdeI cleavage sites at the 3' end of upstream fragment and 5'end of the *rne*$^{E.c.\mathrm{ts}}$ coding sequence. Therefore the nucleotide at position -1 is substituted (A→T), but the ATG of *rne*$^{\mathrm{R.s}}$ and its distance to native promoter as well as RBS is maintained on suicide plasmid and after recombination in the chromosome. All fragments were subcloned into pJET1.2/blunt cloning vector (Thermo Scientific) and sequenced to ensure the absence of any mutations. Previous to simultaneous ligation into ScaI/XbaI cut pPHU281 [3] upstream- and *rne*$^{E.c.\mathrm{ts}}$-fragments were generated by cutting corresponding pJet plasmids with ScaI/NdeI and NdeI/XbaI respectively. The resulting plasmid was linearized with KpnI and EcoRI before ligation with equally cut down fragments. Finally the spectinomycin resistance cassette derived from plasmid pHP45Ω was inserted into the XbaI and KpnI restriction sites to obtain the suicide vector. Prior to this step the resistance cassette was cut and ligated into the BamHI restriction site of pDrive (Qiagen) due to the lack of suitable XbaI and KpnI restriction sites in pHP45Ω [4]. After transformation of *E. coli* S17-1 [5] the suicide plasmid was transferred to *R. sphaeroides* 2.4.1 [6] by diparental conjugation. Conjugants were selected on agar plates containing spectinomycin. Double crossover was confirmed by PCR using chromosomal DNA isolated from conjugants.

l*acZ* based reporter plasmids. We constructed reporter plasmids with *mraZ*::*lacZ* translational fusion and *mraZ* upstream regions of varying length to verify the absence of additional promoters localised between the UpsM promoter and *mraZ*. Therefore we used the reverse primer MraZ_rep_r, which leads to a translational fusion of 11 N-terminal AS to LacZ, in combination with MraZ_rep_f (resulting fragment contains 376 upstream nucleotides with UpsM locus and promoter), with MraZ188up_rep_f (188 upstream nucleotides) or with MraZ67up_rep_f (67 upstream nucleotides) for PCR. For the construction of a reporter plasmid with *uppM*::*lacZ* translational fusion of 3 N-terminal AS of the peptide we used the primer pair MraZ_rep_f / 0682ORF3_rep_r for PCR. PCR products were subcloned into pJet1.2 (Thermo Scientific) and sequenced to ensure the absence of mutations. After excision with BamHI and HindIII sequenced fragments were ligated with matching restriction sites of pPHU235 [3]. Resulting plasmids pPHUmraZUpsM, pPHUmraZ188up, pPHUmraZ67up and pPHUORF were used to transform *E. coli* S17-1 [7] for a subsequent transfer to *R. sphaeroides* 2.4.1 [6] by diparental conjugation.

Construction of strain *R. sphaeroides* 2.4.1 pBBRUpsMx2. For an overexpression of the small RNA UpsM we amplified the gene locus along with promoter and terminator twice with the two primer pairs KpnI_IGR0682_f / IGR0682_Hind_r and Hind_IGR0682_f / IGR0682_EcoRV_r. Both Fragments were subcloned into pJET1.2/blunt cloning vector (Fermentas), excised with the corresponding restriction enzymes (KpnI+HindIII and HindIII+EcoRV) and ligated into pBBR1MCS-II [8]. After transformation of *E. coli* S17-1, overexpression plasmids as well as the empty plasmid pBBR1MCS-II [8] were transferred to *R. sphaeroides* 2.4.1 by diparental conjugation. Conjugants were selected on agar plates containing kanamycin. Overexpression was verified via Norther blot.

Supplementary References

1. Apirion D. Isolation, genetic mapping and some characterization of a mutation in *Escherichia coli* that affects the processing of ribonuleic acid. Genetics. 1978;90(4):659-71. PubMed PMID: 369943; PubMed Central PMCID: PMC1213911.
2. Goldblum K, Apririon D. Inactivation of the ribonucleic acid-processing enzyme ribonuclease E blocks cell division. J Bacteriol. 1981;146(1):128-32. PubMed PMID: 6163761; PubMed Central PMCID: PMC217061.
3. Hubner P, Willison JC, Vignais PM, Bickle TA. Expression of regulatory *nif* genes in *Rhodobacter capsulatus*. J Bacteriol. 1991;173(9):2993-9. PubMed PMID: 1902215; PubMed Central PMCID: PMC207883.
4. Fellay R, Frey J, Krisch H. Interposon mutagenesis of soil and water bacteria: a family of DNA fragments designed for in vitro insertional mutagenesis of gram-negative bacteria. Gene. 1987;52(2-3):147-54. PubMed PMID: 3038679.
5. Simon R, O'Connell M, Labes M, Puhler A. Plasmid vectors for the genetic analysis and manipulation of rhizobia and other gram-negative bacteria. Methods Enzymol. 1986;118:640-59. PubMed PMID: 3005803.
6. van Niel CB. The Culture, General Physiology, Morphology, and Classification of the Non-Sulfur Purple and Brown Bacteria. Bacteriol Rev. 1944;8(1):1-118. PubMed PMID: 16350090; PubMed Central PMCID: PMC440875.
7. Simon LD, Randolph B, Irwin N, Binkowski G. Stabilization of proteins by a bacteriophage T4 gene cloned in *Escherichia coli*. Proc Natl Acad Sci U S A. 1983;80(7):2059-62. PubMed PMID: 6340113; PubMed Central PMCID: PMC393752.
8. Kovach ME, Elzer PH, Hill DS, Robertson GT, Farris MA, Roop RM, 2nd, et al. Four new derivatives of the broad-host-range cloning vector pBBR1MCS, carrying different antibiotic-resistance cassettes. Gene. 1995;166(1):175-6. PubMed PMID: 8529885.
