# Supplementary material for: The Conserved Dcw Gene Cluster of R. sphaeroides Is Preceded by an Uncommonly Extended 5’ Leader Featuring the sRNA UpsM
Source: PLoS One. 2016 Nov 1;11(11):e0165694. doi: 10.1371/journal.pone.0165694 (PMC5089854; doi:10.1371/journal.pone.0165694)
Supplement: S1 Table — The transcriptome of both strains was compared by microarray analysis during exponential growth under aerobic and non-stress conditions and after 90 min of 1O2 stress. For both conditions a biological duplicate of arrays was hybridized with RNA from three biological independent cultures per strain. A Pearson correlation coefficient between the replica of 0.97 and 0.95 was calculated. Changes in expression levels of protein-coding genes passing the selection criteria of microarray analysis, which is a reliable A-value ≥ 12 and a log2 fold change of > 0.65 or < -0.65 between the two strains, are shown. (DOCX) [file pone.0165694.s009.docx]

Table S1: Gene expression of an UpsM overexpression strain *R. sphaeroides* 2.4.1 pBBRUpsMx2 was analysed in comparison to the strain *R. sphaeroides* 2.4.1 pBBR1MCS2 harbouring the empty vector to get first insights into the biological function of UpsM. The transcriptome of both strains was compared by microarray analysis during exponential growth under aerobic and non-stress conditions and after 90 min of ^1^O_2_ stress. For both conditions a biological duplicate of arrays was hybridized with RNA from three biological independent cultures per strain. A Pearson correlation coefficient between the replica of 0.97 and 0.95 was calculated. Changes in expression levels of protein-coding genes passing the selection criteria of microarray analysis, which is a reliable A-value ≥ 12 and a log2 fold change of > 0.65 or < -0.65 between the two strains, are shown.

| **RSP no.** | **Gene** | **Description** | **Ratio**  **[log2 FC]** |
| --- | --- | --- | --- |
| **Gene expression of strain *R. sphaeroides* 2.4.1 pBBRUpsMx2 vs. *R. sphaeroides* 2.4.1 pBBR1MCS2 (aerobic growth)** | | | |
| RSP_1741 |  | Possible LuxR family protein | -0.84 |
| RSP_3622 |  | hypothetical protein | -0.77 |
| RSP_2346 |  | Cold-shock DNA-binding domain protein | -0.76 |
| RSP_1517 | spbA | Histone-like protein of HNS family | -0.74 |
| RSP_6078 |  | hypothetical protein | -0.73 |
| RSP_3539 |  | Hemolysin-type calcium-binding region, RTX | -0.72 |
| RSP_1951 |  | hypothetical protein | -0.71 |
| RSP_3621 |  | Cold-shock DNA-binding protein | -0.70 |
| RSP_2175 |  | ABC transporter, ATPase subunit | -0.70 |
| RSP_2283 | rpsD | 30S ribosomal protein S4 | -0.69 |
| RSP_2536 | rhlE | ATP-dependent helicase, DEAD-box | -0.68 |
| RSP_0965 | sucA | 2-oxoglutarate dehydrogenase E1 component | -0.67 |
| RSP_0819 | rhlE2 | DEAD/DEAH box helicase | -0.66 |
| RSP_1952 |  | Cold-shock DNA-binding domain protein | -0.66 |
| RSP_0626 | infA | translation initiation factor IF-1 | -0.65 |
| RSP_1409 |  | Beta-Ig-H3/Fasciclin | 0.66 |
| RSP_6142 |  | hypothetical protein | 0.66 |
| RSP_4137 | y4bF | Putative transposase | 0.66 |
| RSP_2641 |  | hypothetical protein | 0.69 |
| RSP_1438 |  | ABC Fe hydroxamate (ferrichrome) transporter, inner membrane subunits | 0.70 |
| RSP_3056 |  | TonB dependent-iron siderophore receptor | 0.70 |
| RSP_2292 |  | hypothetical protein | 0.72 |
| RSP_2557 | asnB | putative asparagine synthetase( EC:6.3.5.4 ) | 0.76 |
| RSP_7203 |  | hypothetical protein | 0.77 |
| RSP_3945 |  | Carbohydrate kinase, PfkB | 0.78 |
| RSP_0339 |  | hypothetical protein | 0.85 |
| RSP_2308 |  | hypothetical protein | 0.85 |
| RSP_1016 |  | small heat shock protein | 0.88 |
| RSP_1063 |  | Putative Cyclic-diGMP cyclase/phophodiesterase | 0.90 |
| RSP_3303 | mcpG | Putative methyl accepting chemotaxis protein, McpG | 0.96 |
| RSP_1243 |  | transcriptional regulator, LacI family | 0.99 |
|  | | | |
| **Gene expression of strain *R. sphaeroides* 2.4.1 pBBRUpsMx2 vs. *R. sphaeroides* 2.4.1 pBBR1MCS2 (90 min ^1^O_2_ stress)** | | | |
| RSP_0908 | sitD | ABC Mn/Fe transporter, inner membrane subunit SitD | -1.05 |
| RSP_0906 | sitC | ABC Mn/Fe transporter, inner membrane subunit SitC | -1.04 |
| RSP_0904 | sitA | ABC Mn/Fe transporter, periplasmic substrate-binding protein SitA | -0.85 |
| RSP_1409 |  | Beta-Ig-H3/Fasciclin | -0.84 |
| RSP_0905 | sitB | ABC Mn/Fe transporter, ATPase subunit SitB | -0.80 |
| RSP_2358 |  | possible phage phi-C31 gp36-like protein / capsid protein, HK97 family | -0.78 |
| RSP_1829 | coxIII | Cytochrome c oxidase, aa3-type, subunit III( EC:1.9.3.1 ) | -0.70 |
| RSP_1517 | spbA | Histone-like protein of HNS family | -0.69 |
| RSP_2718 |  | possible outer membrane protein | -0.66 |
| RSP_1613 |  | TRAP-T family transporter, DctP subunit | -0.65 |
| RSP_2012 |  | NUDIX hydrolase, MutT | 0.65 |
| RSP_3056 |  | TonB dependent-iron siderophore receptor | 0.67 |
| RSP_3278 |  | Von Willebrand domain containing protein | 0.71 |
| RSP_1438 |  | ABC Fe hydroxamate (ferrichrome) transporter, inner membrane subunits | 0.72 |
| RSP_3764 |  | hypothetical protein | 0.73 |
| RSP_3566 |  | dimethylglycine dehydrogenase | 0.74 |
| RSP_2641 |  | hypothetical protein | 0.75 |
| RSP_0620 |  | hypothetical protein | 0.75 |
| RSP_4137 | y4bF | Putative transposase | 0.76 |
| RSP_7203 |  | hypothetical protein | 0.80 |
| RSP_0339 |  | hypothetical protein | 0.82 |
| RSP_3303 | mcpG | Putative methyl accepting chemotaxis protein, McpG | 0.83 |
| RSP_2308 |  | hypothetical protein | 0.84 |
| RSP_1016 |  | small heat shock protein | 0.85 |
| RSP_1243 |  | transcriptional regulator, LacI family | 0.88 |
| RSP_1063 |  | Putative Cyclic-diGMP cyclase/phophodiesterase | 1.05 |
