# Supplementary material for: The Conserved Dcw Gene Cluster of R. sphaeroides Is Preceded by an Uncommonly Extended 5’ Leader Featuring the sRNA UpsM
Source: PLoS One. 2016 Nov 1;11(11):e0165694. doi: 10.1371/journal.pone.0165694 (PMC5089854; doi:10.1371/journal.pone.0165694)
Supplement: S2 Table — (DOCX) [file pone.0165694.s010.docx]

**S2 Table. Strains and plasmids used in this study**

| **Strains/Plasmids** | **Description** | **Source / Reference** |
| --- | --- | --- |
| ***Rhodobacter sphaeroides*** | | |
| 2.4.1 | Wild type | [1] |
| Δhfq | Δ(hfq::ΩSp) | [2] |
| rne^E.coli(ts)^ | Δ(rne::rne^E.c.ts^;Sp) | This study |
| Δrnc | Δ(rnc::Km) | [3] |
| Δrnj | Δ(rnj::Km) | [4] |
| ΔrpoHI | Δ(rpoHI::Km) | [5] |
| ΔrpoHII | Δ(rpoHII::ΩSp) | [6] |
| ΔrpoHI/II | Δ(rpoHI::Km) Δ(rpoHII::ΩSp) | [5] |
| ***E.coli*** | | |
| S17-1 | *recA pro hsdR* RP4-2-Tc::Mu-Km::Tn7 *tra^+^* Km^r^, Sp^r^ | [7] |
| JM109 | *recA1 supE44 endA1 hsdR17 gyrA96 relA1 thi (lac–proAB)* | New England Biolabs |
| N3431 | *Hfr(PO1), lacZ43(Fs), λ-, rne-3071(ts), relA1, spoT1, thiE1* | [8, 9] |
| **Plasmids** | | |
| pDrive | Ap^r^, Km^r^ cloning vector | Qiagen |
| pJET1.2 | Ap^r^, cloning vector | Thermo Scientific |
| pPHU281 | Tc^r^, suicide vector for *R. sphaeroides* | [10] |
| pHP45ΩSp | Sp^r^, source for Sp cassette with terminator | [11] |
| pPHU*rne*::rne^E.coli(ts)^ΩSp | Tc^r^, pPHU281 containing upstream and downstream of *rne* with *rne-3071(ts*) and pHP45ΩKm derived Km^r^ | This study |
| pPHU235 | Tc^r^, broad-host-range *lacZ* fusion vector | [10] |
| pPHU4352 | Tc^r^, pPHU235 containing 16S rRNA promoter | [12] |
| pPHUmraZUpsM | Tc^r^, pPHU235 containing UpsM *mraZ* fragment for *lacZ* fusion | This study |
| pPHUmraZ188up | Tc^r^, pPHU235 containing UpsM *mraZ* fragment for *lacZ* fusion | This study |
| pPHUmraZ67up | Tc^r^, pPHU235 containing *mraZ* fragment for *lacZ* fusion | This study |
| pPHUORF | Tc^r^, pPHU235 containing *upsP* fragment for *lacZ* fusion | This study |
| pBBR1MCS2 | Km^r^, broad-host-range cloning vector | [13] |
| pBBRUpsMx2 | Km^r^, pBBR1MCS2 containing two copies of the UpsM locus | This study |

1. van Niel CB. The Culture, General Physiology, Morphology, and Classification of the Non-Sulfur Purple and Brown Bacteria. Bacteriol Rev. 1944;8(1):1-118. PubMed PMID: 16350090; PubMed Central PMCID: PMC440875.

2. Glaeser J, Zobawa M, Lottspeich F, Klug G. Protein synthesis patterns reveal a complex regulatory response to singlet oxygen in *Rhodobacter*. J Proteome Res. 2007;6(7):2460-71. doi: 10.1021/pr060624p. PubMed PMID: 17536848.

3. Rische-Grahl T, Weber L, Remes B, Forstner KU, Klug G. RNase J is required for processing of a small number of RNAs in *Rhodobacter sphaeroides*. RNA Biol. 2014;11(7):855-64. doi: 10.4161/rna.29440. PubMed PMID: WOS:000342901600012.

4. Rische T, Klug G. The ordered processing of intervening sequences in 23S rRNA of Rhodobacter sphaeroides requires RNase J. RNA Biol. 2012;9(3):343-50. doi: 10.4161/rna.19433. PubMed PMID: 22336705.

5. Nuss AM, Glaeser J, Berghoff BA, Klug G. Overlapping alternative sigma factor regulons in the response to singlet oxygen in *Rhodobacter sphaeroides*. J Bacteriol. 2010;192(10):2613-23. doi: 10.1128/JB.01605-09. PubMed PMID: 20304993; PubMed Central PMCID: PMC2863570.

6. Nuss AM, Glaeser J, Klug G. RpoH(II) activates oxidative-stress defense systems and is controlled by RpoE in the singlet oxygen-dependent response in *Rhodobacter sphaeroides*. J Bacteriol. 2009;191(1):220-30. doi: 10.1128/JB.00925-08. PubMed PMID: 18978062; PubMed Central PMCID: PMC2612413.

7. Simon LD, Randolph B, Irwin N, Binkowski G. Stabilization of proteins by a bacteriophage T4 gene cloned in *Escherichia coli*. Proc Natl Acad Sci U S A. 1983;80(7):2059-62. PubMed PMID: 6340113; PubMed Central PMCID: PMC393752.

8. Apirion D. Isolation, genetic mapping and some characterization of a mutation in *Escherichia coli* that affects the processing of ribonuleic acid. Genetics. 1978;90(4):659-71. PubMed PMID: 369943; PubMed Central PMCID: PMC1213911.

9. Goldblum K, Apririon D. Inactivation of the ribonucleic acid-processing enzyme ribonuclease E blocks cell division. J Bacteriol. 1981;146(1):128-32. PubMed PMID: 6163761; PubMed Central PMCID: PMC217061.

10. Hubner P, Willison JC, Vignais PM, Bickle TA. Expression of regulatory *nif* genes in *Rhodobacter capsulatus*. J Bacteriol. 1991;173(9):2993-9. PubMed PMID: 1902215; PubMed Central PMCID: PMC207883.

11. Fellay R, Frey J, Krisch H. Interposon mutagenesis of soil and water bacteria: a family of DNA fragments designed for in vitro insertional mutagenesis of gram-negative bacteria. Gene. 1987;52(2-3):147-54. PubMed PMID: 3038679.

12. Mank NN, Berghoff BA, Hermanns YN, Klug G. Regulation of bacterial photosynthesis genes by the small noncoding RNA PcrZ. Proc Natl Acad Sci U S A. 2012;109(40):16306-11. doi: 10.1073/pnas.1207067109. PubMed PMID: 22988125; PubMed Central PMCID: PMC3479615.

13. Kovach ME, Elzer PH, Hill DS, Robertson GT, Farris MA, Roop RM, 2nd, et al. Four new derivatives of the broad-host-range cloning vector pBBR1MCS, carrying different antibiotic-resistance cassettes. Gene. 1995;166(1):175-6. PubMed PMID: 8529885.
