# Supplementary material for: The Conserved Dcw Gene Cluster of R. sphaeroides Is Preceded by an Uncommonly Extended 5’ Leader Featuring the sRNA UpsM
Source: PLoS One. 2016 Nov 1;11(11):e0165694. doi: 10.1371/journal.pone.0165694 (PMC5089854; doi:10.1371/journal.pone.0165694)
Supplement: S3 Table — (DOCX) [file pone.0165694.s011.docx]

| **Oligonucleotide** | **Sequence** | **Purpose** | **Source** |
| --- | --- | --- | --- |
|  |  |  |  |
| p-5S | 5'-CTTGAGACGCAGTACCATTG-3' | probe | [1] |
| p-0682 (UpsM) | 5'-GACTCAGGTGGTCGCCAGATACC-3' | probe | This study |
| *gloB*_A | 5′-GAACAATTACGCCTTCTC-3′ | test PCR for DNA | [2] |
| *gloB*_B | 5´-CATCAGCTGGTAGCTCTC-3′ | test PCR for DNA | [2] |
| MraZ_rep_f | 5´-CAGGTTGTGCGGATCCAACTTG-3′ | PCR | This study |
| MraZ67up_rep_f | 5´-GGATCCCTTTCGTTTCCGGGGTGC-3′ | PCR | This study |
| MraZ188up_rep_f | 5´-GGATCCAATGACAGATCCGGCG-3′ | PCR | This study |
| MraZ_rep_r | 5´-CTTGGCGTCAAGCTTCTGGTTG-3′ | PCR | This study |
| 0682ORF3_rep_r | 5´-AAGCTTATCTGTCATTGGATGAAAGCGGG-3' | PCR | This study |
| R.s.rneUP_f | 5´-AGTACTCGGATCTCTATTACCGGCTCG-3' | PCR | This study |
| R.s.rneUP_r | 5´-CATATGATTCCATGGCACCCG-3' | PCR | This study |
| R.s.rneDOWN_f | 5´-GGTACCGCTGATCTCCAGAGATTGACAC-3' | PCR | This study |
| R.s.rneDOWN_r | 5´-GAATTCTGCATTGGACAGGAAGAAGG-3' | PCR | This study |
| E.c.rne_fw | 5´-CATATGAAAAGAATGTTAATCAACGCAAC-3' | PCR | This study |
| E.c.rne_rev | 5´-TCTAGAAACTGGGTATGTTTTGTCTGCC-3' | PCR | This study |
| KpnI_IGR0682_f | 5´-GGTACCGAGCCACCTTCTTCCCATGGC-3' | PCR | This study |
| IGR0682_Hind_r | 5´-AAGCTTCACTGTCCGGTGCCGTTC-3' | PCR | This study |
| Hind_IGR0682_f | 5´-AAGCTTGAGCCACCTTCTTCCCATGGC-3' | PCR | This study |
| IGR0682_EcoRV_r | 5´-GATATCTTCACTGTCCGGTGCCGTTC-3' | PCR | This study |
| pUpsM_A | 5´-CGAGATGAGAACGGGACAG-3' | qRT-PCR | This study |
| pUpsM_B | 5´-ATCGCTGGGTGTCCAACT-3' | qRT-PCR/5’ RACE | This study |
| pUpsM_mraZ_A | 5´-CAGTGGCAGCCGCTCGAT-3' | qRT-PCR | This study |
| pUpsM_mraZ_B | 5´-GCGCCTTGGCGTCAACCT-3' | qRT-PCR/5’ RACE | This study |
| pmraZ_A | 5´-AGAGGCGAATACAACCAG-3' | qRT-PCR | This study |
| pmraZ_B | 5´-TAGCATTCGACGTAGGAG-3' | qRT-PCR | This study |
| 16S_A | 5´-CCTGGAACTGCCTTTGAAAC-3' | qRT-PCR | This study |
| 16S_B | 5´-GCGGAATGCTTAATCCGTTA-3' | qRT-PCR | This study |
| Oligo dT-anchor Primer | 5´-GACCACGCGTATCGATGTCGAC  TTTTTTTTTTTTTTTTV-3' (V=A,C or G) | 5’ RACE | Roche |
| PCR anchor Primer | 5´-GACCACGCGTATCGATGTCGAC-3' | 5’ RACE | Roche |

**S3 Table. Oligonucleotides used in this study.**

1. Berghoff BA, Glaeser J, Sharma CM, Vogel J, Klug G. Photooxidative stress-induced and abundant small RNAs in *Rhodobacter sphaeroides*. Mol Microbiol. 2009;74(6):1497-512. doi: 10.1111/j.1365-2958.2009.06949.x. PubMed PMID: 19906181.

2. Glaeser J, Klug G. Photo-oxidative stress in *Rhodobacter sphaeroides*: protective role of carotenoids and expression of selected genes. Microbiology. 2005;151(Pt 6):1927-38. doi: 10.1099/mic.0.27789-0. PubMed PMID: 15942000.
